# Towards bridging the digital divide: training healthcare professionals for digitally inclusive healthcare systems

**DOI:** 10.1186/s41256-025-00433-x

**Published:** 2025-07-30

**Authors:** Efrat Neter, Max J. Western, Rosie Cooper, Anabelle Macedo Silva, Laura M. König

**Affiliations:** 1 Ruppin Academic Center, Emek Hefer, Israel; 2 Centre for Motivation and Behaviour Change, Department for Health, University of Bath, Bath, UK; 3 Nuffield Department of Primary Care Health Sciences, University of Oxford, Oxford, UK; 4 Instituto de Estudos em Saúde Coletiva, Universidade Federal do Rio de Janeiro, Rio de Janeiro, Brazil; 5 Leibniz Science Campus on Digital Public Health, Bremen, Germany; 6 Faculty of Life Sciences: Food, Nutrition and Health, University of Bayreuth, Bayreuth, Germany; 7 Faculty of Psychology, University of Vienna, Vienna, Austria

## Abstract

Over the past decade, the integration of information and communications technologies (ICTs) in healthcare has grown significantly, as has the rapid increase in internet access and mobile device ownership by individuals. However, challenges such as the digital divide, encompassing disparities in access, usage skills, and the benefits derived from ICT use, persist. Addressing this divide is crucial for maximizing the potential of digital health technologies, particularly for more vulnerable people in society who often require the most support. This commentary paper’s aim is to advocate training, in both educational and healthcare settings, so as to contribute towards bridging the digital divide. We propose that educational programs for healthcare professionals in academic institutions can integrate modules on the digital health divide within existing courses on social determinants of health (e.g., sociology, epidemiology, and health informatics) or in specific courses on digital health. The recommended courses should include modules on the digital divide, its causes, implications, and strategies to first assess and then enhance digital and health literacy among patients. Training healthcare professionals in work settings would be part of continuous professional development. This training should include assessing digital health literacy, identifying barriers to uptake, engagement and impact of digital health tools, and providing tailored education on digital health tools or interventions. Healthcare professionals should follow protocols to ensure the effective use of digital health tools by diverse patients and have access to community resources for ongoing support. Finally, the paper suggests service-wide international standards for ameliorating the digital divide.

## Introduction

Digital health technologies are an enabling factor in achieving better health and well-being and the vision of health for all. The past decade has seen rapid growth in the use of information and communications technologies (ICTs) in healthcare worldwide. The use of adequately designed digital health technologies has the potential to strengthen health systems and even reduce health inequalities, including increased access to and quality of healthcare, supporting healthcare workers in clinical decision-making, and strengthening data management. With the rapid increase in ownership of internet access and mobile devices worldwide, many investments are being made in digital health tools to provide better healthcare. Despite considerable progress, crucial challenges such as the digital divide persist.

The term "digital divide" refers to the gap that exists between individuals or communities who have access (1st digital divide), skills (2nd digital divide), and subsequent benefits from the use of ICTs (labelled 3rd digital divide) versus those who do not have these resources or who have them to a lesser extent [1]. The access divide focuses on disparities in physical access to ICTs, including computers, internet connectivity, mobile devices (e.g., smartphones, tablets), and broadband, with geographic disparities (e.g., rural vs. urban areas) being one example. The usage or skill divide focuses on how people with access to technology utilize it. The usage divide highlights the importance of digital skills and the ability to navigate and make the most digital resources. The third divide is the benefits or empowerment extracted from digital use [1]. These apply to an enhanced ability to engage in decision-making processes, voice one's opinions, and access vital services, such as healthcare or government information. As digitization permeates all levels of determinants of health inequities, exposure to digital technology and digitally mediated content”constitute[s] a health-protective factor … or a health risk factor”, depending on the content [2]. Moreover, recent research has suggested that access to and engagement in studies examining digital health interventions (DHIs) not only reflects existing offline social inequality [3] but also widens the gap in health disparities (e.g., eligibility criteria and recruitment strategies limit study samples and inadvertently limit generalizability) [4].

Healthcare professionals (HCPs) are important gatekeepers for DHIs: they may raise awareness about existing digital tools, provide access to relevant technology, or train patients in how to use it adequately to maximize engagement and benefits [5]. Moreover, HCPs often hold the trust of citizens [6], who assume HCPs will introduce or prescribe them an effective and up-to-date DHI, which will also guard their privacy [7]. However, many HCPs feel ill-equipped for this task; they indicate concerns regarding data protection and an inability to distinguish good tools from bad ones. Conversely, HCPs may hold stereotypes regarding which patients might (not) be able to use DHIs, e.g., older adults, which is not necessarily true [8]. The potential of DHIs and barriers to its realization underscore the need for appropriate training of HCPs to overcome these barriers. On the basis of discussions held during an international expert workshop [4], the present paper aims to propose how HCPs, in training and practice, can be educated about the digital divide in their field, specifically in DHIs, and how to develop skills in drawing more people from the “have-nots” into the 'haves" in DHI engagement. The following focuses on policies of training in educational setting and healthcare services as a vehicle to address the digital divide.

## Proposed training policies on the digital health divide

### Educational system

The educational system is a natural place to train and equip professionals. Many training programs for HCPs already include a module on social determinants, which would be a natural starting point for discussing the digital health divide. Indeed, the digital health divide could be addressed in courses teaching sociology, epidemiology, health informatics, health administration, health policy, or public health. Moreover, HCP training programs, such as nursing [9], are starting to include specific courses on digital health [10]; their initial implementation could be viewed as an opportunity to shape the content of such courses or modules by specifically addressing the digital divide.

We propose that courses on digital health should include a module on the digital divide, including definitions, causes, and implications for health and society, and ways to reduce the digital divide. Students should first be taught and provided with tools to assess digital literacy and digital health literacy and then educate patients on the benefits of DHIs and the importance of demonstrating to patients the use of DHIs and providing ongoing support to patients who begin to use DHIs (e.g., follow-up appointments to monitor progress, availability to answer questions or concerns). Such a module could use simulations. A recent example is a learning objective targeting diversity and equity in a course on a digital service using wearables [11]. The proposed modules address the second digital divide.

### Workplace

Digital services are an inseparable part of healthcare today, and contemporary workplaces continuously educate their employees in adopting new technologies and upgrading their digital competencies, as this is a prerequisite for many work activities. This training should be provided through continuous professional development (CPD) programs, either carried out locally and in-person or aided by knowledge/learning management systems.

Training HCPs on how to adjust their practices to reduce digital disparities is essential for bringing in and engaging more patients in the use of DHIs [12]. For example, a physiotherapist should go beyond assigning a mobile application for exercising movements in the patient's home environment. Rather, physiotherapists should be guided by a protocol that sets standards for professional conduct and does not leave the interaction to variations in HCPs’ motivation and skills. The protocol, for example, should instruct HCPs to assess the digital health literacy of patients, identify barriers to using DHIs, provide education and training on the specific DHIs, and then guide patients through a series of practice exercises. Materials should be available in different languages and language registers. Finally, HCPs should have at their disposal a list of community organizations/groups that can assist in ongoing engagement with digital health tools among patients from various backgrounds. This proposed training addresses the second digital divide of skill, with a potential of ameliorating the third digital divide of benefits.

## Next steps in promoting education about the digital health divide globally

**Fig. 1:**
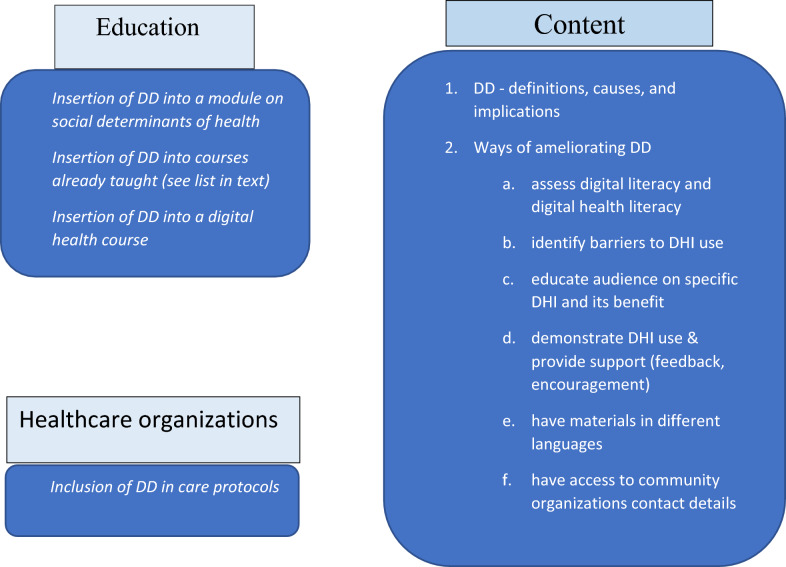
Recommendations for the education and training of healthcare professionals on the digital divide (DD) and its amelioration

A necessary first step is to assess and compare existing HCP training curricula on DHIs and associated inequalities. Once this understanding is developed, we recommend devising comprehensive training and professional guidelines to aid HCPs in assessing their patients’ (and/or their caregivers’) access to DHIs in terms of digital literacy, access to suitable devices (e.g., smartphones or computers), internet connectivity, and motivation (see education and training recommendations in Fig. 1). We suggest that this should incorporate training to identify barriers patients may face in DHI access or adoption, and then develop strategies and means for patients to overcome these obstacles. Additionally, any solution must be culturally appropriate for the patient/caregiver; therefore, training must also focus on developing cultural competencies and sensitivities. The above addresses both the first and second digital divide.

Recognizing the dynamic nature of DHIs, we propose that further research should focus on the needs of HCPs to keep their skills and knowledge updated and enable them to practice safely and effectively. This includes not only staying updated with emerging technologies but also developing competencies related to patient assessment and addressing barriers to DHI adoption. As the digital health divide is not limited to just patient groups, it should not be assumed that HCPs all have high-level knowledge and competencies for digital technologies in equal measure. Therefore, future research could explore the adequacy of existing courses and/or CPD, and develop new materials (e.g., online workshops, conferences).

Creating service-wide international standards for ameliorating the digital divide through HCP education and training can set an agenda and transform a vision into policies, which admittedly need to be continuously revised to accommodate technological changes as well as usher in competitiveness, responsiveness, and innovation (which are not the focus of the present position paper). The education and training of HCPs should be part of the global strategy to realize the potential of digital services in an accessible, scalable, and equitable manner [13] and benefit from global resolutions, strategies, commitment, policies (e.g., regulation) and investment of governments and organizations. Realizing the potential of digital services could mitigate the third digital divide (i.e., accrued benefits by geography, age, income and education, etc.) and advance the United Nations Sustainability Goal 10 (reduced inequalities).

Taking this path should be accompanied by a research agenda examining the engagement of citizens with DHIs by indicators of inequality, as well as efficacy and effectiveness of DHIs by these indicators of inequality [14]. As delineated above, König et al. pointed out that research itself inadvertently induces inequality through such practices as eligibility criteria, recruitment strategies or analytic decisions [4], and called for awareness by researchers and for research policies mandating inequality indicators into reporting guidelines and pre-registration templates [15]. Indeed, ensuring both researchers and HCPs acknowledge and work to address the digital divide when developing, evaluating, and applying DHIs will be      paramount for a thriving, technologically-enhanced, healthcare landscape.

## Data Availability

Nonapplicable (due to manuscript type).

## Abbreviations

CPD: Continuous professional development
DD: Digital divide
DHI: Digital health interventions
HCPs: Healthcare professionals
ICT: Information and communication technologies

## References

[1] Lyles CR, Nguyen OK, Khoong EC, Aguilera A, Sarkar U. Multilevel determinants of digital health equity: a literature synthesis to advance the field. Annual Rev Public Health. 2023;44(1):383–405. 10.1146/annurev-publhealth-071521-023913. 36525960 10.1146/annurev-publhealth-071521-023913 PMC10329412

[2] Jahnel T, Dassow HH, Gerhardus A, Schüz B. The digital rainbow: digital determinants of health inequities. Digital Health. 2022;8:20552076221129092. 10.1177/20552076221129093. 36204706 10.1177/20552076221129093 PMC9530552

[3] Van Kessel R, Seghers LE, Anderson M, Schutte NM, Monti G, Haig M, et al. A scoping review and expert consensus on digital determinants of health. Bull World Health Organ. 2024;2(103):110. 10.2471/BLT.24.292057 PMC11774227 39882497

[4] König LM, Krukowski RA, Kuntsche E, Busse H, Gumbert L, Gemesi K, Western MJ. Reducing intervention-and research-induced inequalities to tackle the digital divide in health promotion. Int J Equity Health. 2023;22(1):249. 10.1186/s12939-023-02055-6. 38049789 10.1186/s12939-023-02055-6 PMC10696856

[5] König LM, Attig C, Franke T, Renner B. Barriers to and facilitators for using nutrition apps: systematic review and conceptual framework. JMIR Mhealth Uhealth. 2021;9(6):e20037. 34254938 10.2196/20037 PMC8409150

[6] Adjekum A, Blasimme A, Vayena E. Elements of trust in digital health systems: scoping review. J Med Int Res. 2018;20(12):e11254. 10.2196/11254 PMC6315261 30545807

[7] Guckert M, Milanovic K, Hannig J, Simon D, Wettengl T, Evers D, Pitt J. The disruption of trust in the digital transformation leading to health 40. Front Digital Health. 2022;4:815573. 10.3389/fdgth.2022.815573 PMC8995643 35419559

[8] Mannheim I, van Zaalen Y, Wouters EJ (2022) Ageism in applying digital technology in healthcare: Implications for adoption and actual use. In: Helena Hirvonen, Mia Tammelin RH, Wouters and EJM, editors. Digital Transformations in Care for Older People. Taylor & Francis p 72–90.

[9] Nazeha N, Pavagadhi D, Kyaw BM, Car J, Jimenez G, Car LT. A digitally competent health workforce: scoping review of educational frameworks. J Med Int Res. 2020;22(11):e22706. 10.2196/22706 PMC7677019 33151152

[10] Sarvesh S, Petula J, Ewe R (2022) Digital health must be better integrated into medical education. BMJ. 376. 10.1136/bmj.o363 35144964

[11] Ward MP, Malloy JS, Kannmacher C, Steinhubl SR. Educating the healthcare workforce of the future: lessons learned from the development and implementation of a ‘Wearables in Healthcare’ course. NPJ Digit Med. 2023;6(1):1–5. 37990139 10.1038/s41746-023-00964-y PMC10663572

[12] Isidori V, Diamanti F, Gios L, Malfatti G, Perini F, Nicolini A, Gaudino A. Digital technologies and the role of health care professionals: Scoping review exploring nurses’ skills in the digital era and in the light of the COVID-19 pandemic. JMIR Nurs. 2022;5(1):e37631. 36194466 10.2196/37631 PMC9579937

[13] World Health Organization. Global Strategy on Digital Health 2020–2025 [Internet]. World Health Organization; 2021. Available from: https:// www.who.int/docs/default-source/documents/gs4dhdaa2a9f352b0445bafbc 79ca799dce4d.pdf

[14] O’Neill J, Tabish H, Welch V, Petticrew M, Pottie K, Clarke M, et al. Applying an equity lens to interventions: using PROGRESS ensures consideration of socially stratifying factors to illuminate inequities in health. J Clin Epidemiol. 2014;67(1):56–64. 24189091 10.1016/j.jclinepi.2013.08.005

[15] Gültzow T, Neter E, & Zimmermann H (2023) Making research look like the world looks: introducing The'inclusivity & diversity add-on for preregistration forms' developed during an EHPS2022 Pre-Conference Workshop. European Health Psychologist, 23(4)..

